# Supplementary figures and images for: Eosinophil Responses at the Airway Epithelial Barrier during the Early Phase of Influenza a Virus Infection in C57BL/6 Mice
Source: Cells. 2021 Feb 27;10(3):509. doi: 10.3390/cells10030509 (PMC7997358; doi:10.3390/cells10030509)

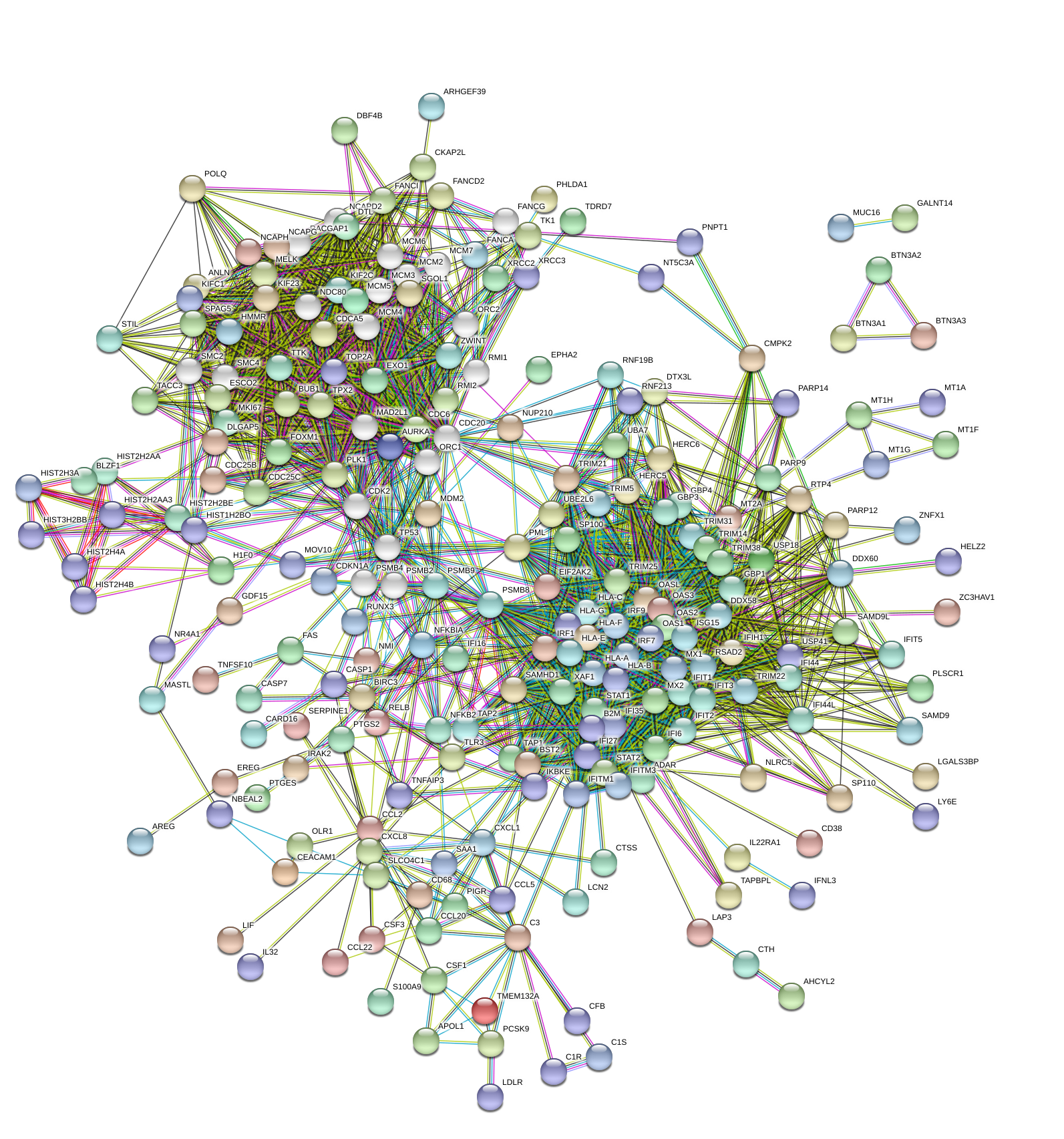

Supplement: Supplementary file 1 [file cells-10-00509-s001.zip › Figures 03.01/FigS1_string_normal_image.png]

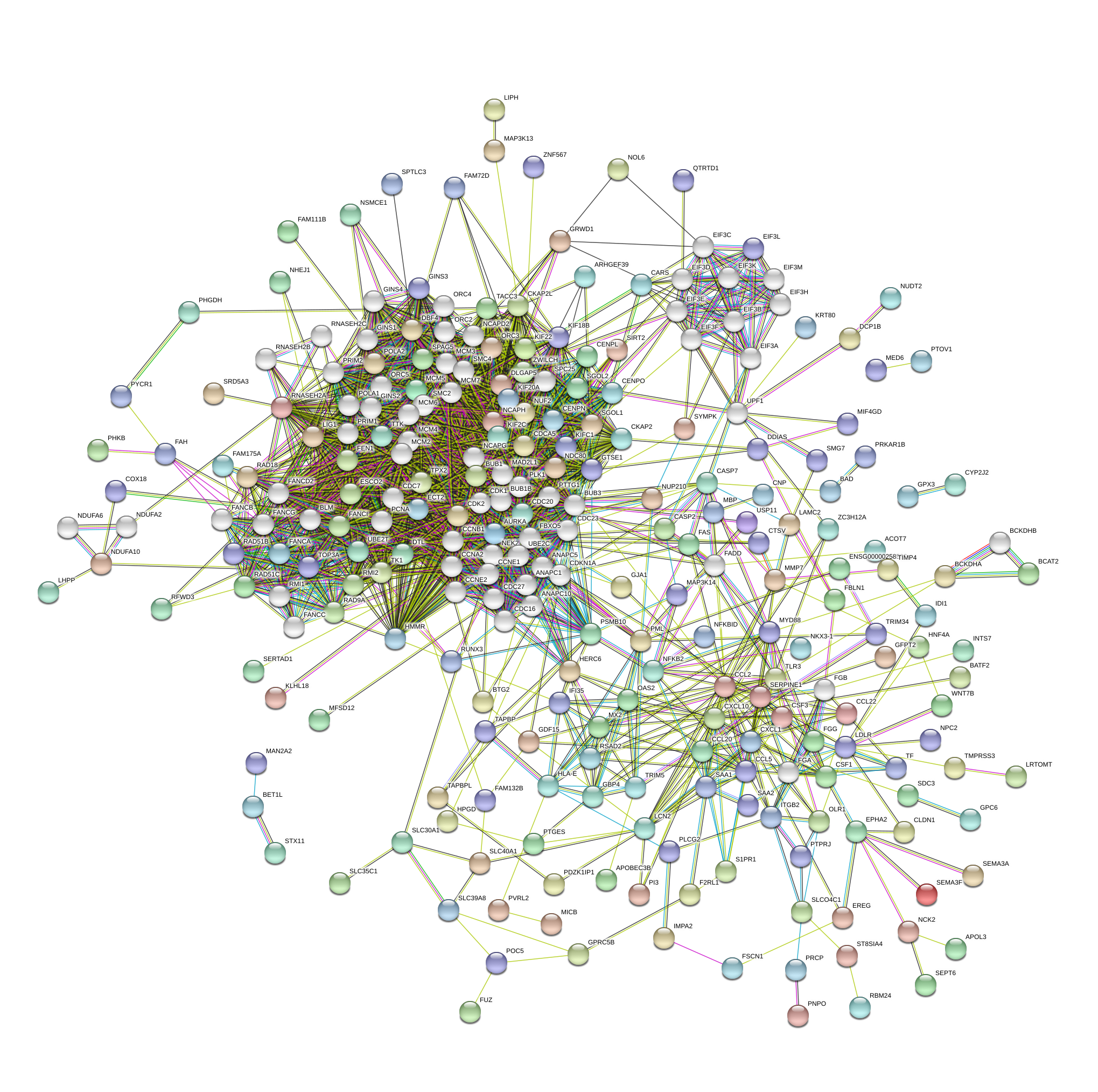

Supplement: Supplementary file 1 [file cells-10-00509-s001.zip › Figures 03.01/FigS2_string_normal_image.png]

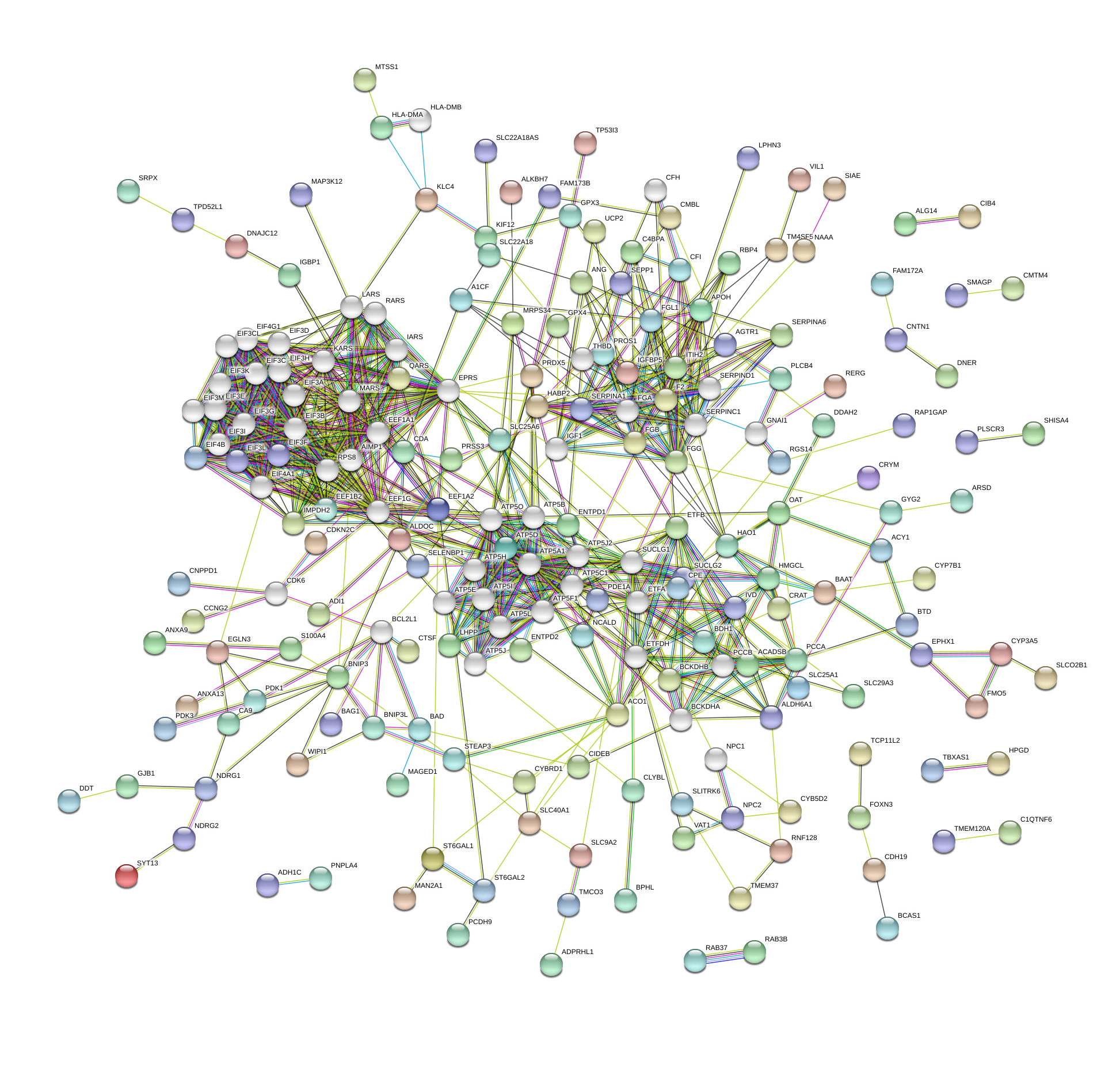

Supplement: Supplementary file 1 [file cells-10-00509-s001.zip › Figures 03.01/FigS3_string_normal_image.png]

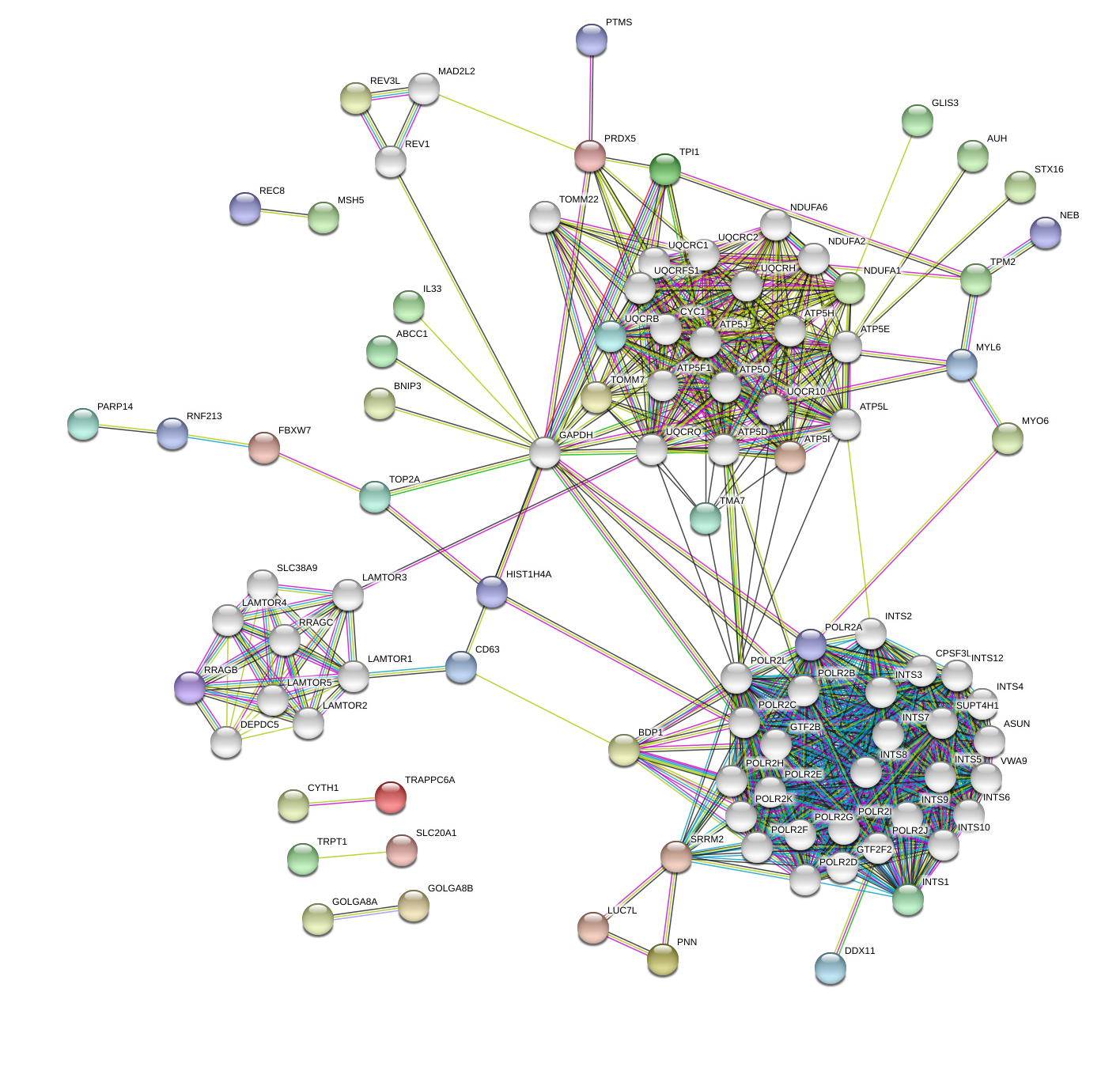

Supplement: Supplementary file 1 [file cells-10-00509-s001.zip › Figures 03.01/FigS4_string_normal_image.png]
